# Effects of unweighting on gait kinematics during walking on a lower-body positive-pressure treadmill in patients with hip osteoarthritis

**DOI:** 10.1186/s12891-020-03909-8

**Published:** 2021-01-08

**Authors:** Yoshiaki Kataoka, Tomohiro Shimizu, Ryo Takeda, Shigeru Tadano, Yuki Saito, Satoshi Osuka, Tomoya Ishida, Mina Samukawa, Tohru Irie, Daisuke Takahashi, Norimasa Iwasaki, Harukazu Tohyama

**Affiliations:** 1 Faculty of Health Sciences, Hokkaido University, Kita 12, Nishi 5, Kita-ku, Sapporo, 060-0812 Japan; 2 Department of Rehabilitation, Health Sciences University of Hokkaido Hospital, 2-5 Ainosato, Kita-ku, Sapporo, 002-8072 Japan; 3 Department of Orthopaedic Surgery, Faculty of Medicine and Graduate School of Medicine, Hokkaido University, Kita 15, Nishi 7, Kita-ku, Sapporo, Hokkaido 060-8638 Japan; 4 Faculty of Engineering, Hokkaido University, Kita 12, Nishi 8, Kita-ku, Sapporo, 060-8628 Japan

**Keywords:** Osteoarthritis, Walking, Weight-bearing, Gait, Hip joint, Pain

## Abstract

**Background:**

Hip osteoarthritis (OA) is a musculoskeletal condition that makes walking difficult due to pain induced by weight-bearing activities. Treadmills that support the body weight (BW) reduce the load on the lower limbs, and those equipped with a lower-body positive-pressure (LBPP) device, developed as a new method for unweighting, significantly reduce pain in patients with knee OA. However, the effects of unweighting on gait kinematics remain unclear in patients with hip OA. Therefore, we investigated the effects of unweighting on kinematics in patients with hip OA during walking on a treadmill equipped with an LBPP device.

**Methods:**

A total of 15 women with hip OA and 15 age-matched female controls wore a three-dimensional (3-D) motion analysis system and walked at a self-selected speed on the LBPP treadmill. Data regarding self-reported hip pain using a numeric rating scale (NRS) in which the scores 0 and 10 represented no pain and the worst pain, respectively, under three different BW conditions (100, 75, and 50%) were collected. Moreover, 3-D peak joint angles during gait under each condition were calculated and compared.

**Results:**

In the hip OA group, the NRS pain scores at 50 and 75% BW conditions significantly decreased compared with that at 100% BW condition (50%, *P* = 0.002; 75%, *P* = 0.026), and the peak hip extension angle decreased compared with that in the healthy controls (*P* = 0.044). In both groups, unweighting significantly decreased the peak hip (*P* < 0.001) and knee (*P* < 0.001) flexion angles and increased the peak ankle plantar flexion angle (*P* < 0.001) during walking.

**Conclusions:**

Unweighting by the LBPP treadmill decreased pain in the hip OA group but did not drastically alter the gait kinematics compared with that in the control group. Therefore, regarding the use of the LBPP treadmill for patients with hip OA, clinicians should consider the benefits of pain reduction rather than the kinematic changes.

## Background

Walking exercises are widely used in individuals with hip osteoarthritis (OA) for rehabilitation [1, 2]. However, these individuals often have difficulty in walking due to pain and excessive force induced by weight-bearing activities. Treadmills equipped with a lower-body positive-pressure (LBPP) device have been developed to provide precise unweighting during walking [3, 4]. Because LBPP treadmills reduce the stress induced by ground reaction forces on the lower limbs, unweighting by the LBPP treadmill has shown to significantly reduce pain in patients with OA and, therefore, has the potential to maintain or enhance aerobic exercise capacity [5, 6]. In addition, LBPP treadmills reduce the load on the cardiopulmonary function [3], thereby reducing the rate of perceived exertion compared with treadmills with a harness system [7].

Investigating gait kinematics on LBPP for hip OA can provide useful information for clinicians when they apply LBPP exercises. However, as a participant’s lower limbs are in a waist-high chamber when using an LBPP treadmill, a conventional motion analysis using an optical method may be difficult, especially for the hip joint, limiting what is known about how unweighting affects gait kinematics. Because of the advances in technology, a wearable-sensor-based three-dimensional (3-D) motion analysis system, which can analyze gait kinematics by seven sensors that consist of triaxial acceleration and gyro sensors, has recently been developed as a tool to analyze gait kinematics [8]. Hence, we thought that we could calculate gait kinematics in participants with hip OA by this system while walking on an LBPP treadmill.

The present study aimed to investigate the use of wearable sensors with an LBPP treadmill and the unweighting effects on 3-D kinematics in participants with hip OA. The present study hypothesized that (1) the participants with OA would report less pain when unweighted by the LBPP treadmill and (2) all participants would exhibit increased peak hip, knee, and ankle joint angles.

## Methods

### Participants

This study was approved by the institutional review board of our university, and informed consent was obtained from all participants. In total, 15 female participants with hip OA and 15 female healthy controls were recruited. The inclusion criteria of the hip OA group were women who were scheduled to undergo unilateral total hip arthroplasty (THA) for treatment of moderate to severe OA and aged < 85 years. The severity of OA was determined on radiography according to the Kellgren and Lawrence (KL) grade [9] in the participants with hip OA. The exclusion criteria of the hip OA group included a history of (1) immunosuppression or autoimmune deficiency, (2) inflammatory arthritis, (3) local or systemic infections, (4) knee arthritis and/or total knee arthroplasty, or (5) symptomatic spinal cord disease. The Harris hip score, which includes sections on pain, function, absence or presence of deformity, and passive range of motion and is scored from 0 (worst) to 100 (best), was used to evaluate participants with hip OA. None of the healthy controls had a history of bone fracture or surgery in the lower limbs; history of neurological, respiratory, or cardiovascular diseases; musculoskeletal disorders within the past 6 months; or previous history of trauma.

### Gait protocol

**Fig. 1:**
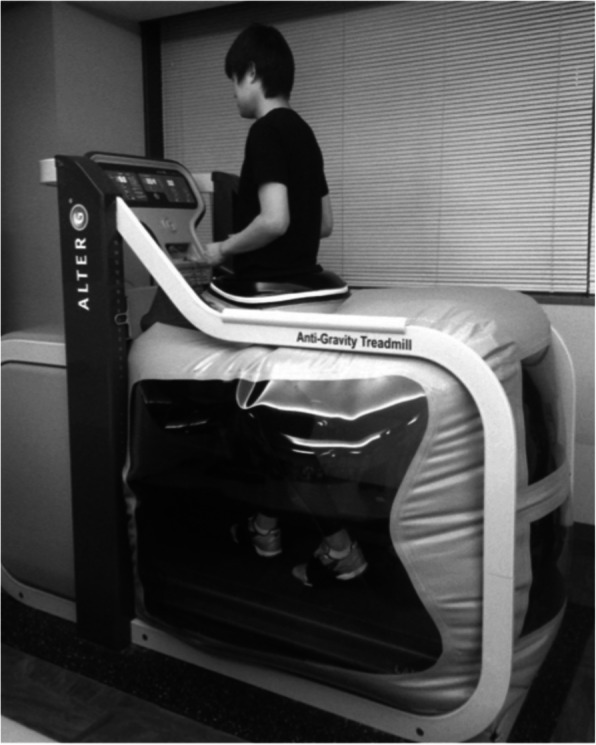
Lower-body positive pressure (LBPP) treadmill. Participants walk on an LBPP treadmill. The positive pressure inflates the chamber to create traction force on the lower limbs

The participants wore specifically designed shorts with sensors while using the LBPP treadmill. The height of the chamber was fixed to accommodate the participant, and sensors from the shorts were attached to the LBPP treadmill. Then, it was set equal to the height of the greater trochanter of the participant’s femur (Fig. 1). To determine the correlation of gravity and the internal pressure of the chamber, calibration was performed for each participant as previously described [10]. The participants walked at a self-selected speed on the LBPP treadmill (Anti-Gravity Treadmill M320, AlterG, Inc., Fremont, California, USA) at 100, 75, and 50% body weight (BW) conditions. The walking speed was consistent across the loading conditions. The participants walked for 30 s under three conditions selected randomly (100, 75, and 50% BW) for the testing procedure. Before recording the walking trials, they were asked to familiarize themselves with walking on the LBPP treadmill for 3 min and given 90 s to adapt to each BW condition. The participants in the hip OA group were asked to assess their hip pain using a numeric rating scale (NRS) in which the scores 0 and 10 represented no pain and the worst pain, respectively [11], during walking under 100, 75, and 50% BW conditions. In addition, participants in the control group walked 30 s again at 100% BW condition to measure the intra-rater reliability results using minimal detectable changes (MDCs) after the gait protocol.

### Data collection using the motion analysis system

**Fig. 2:**
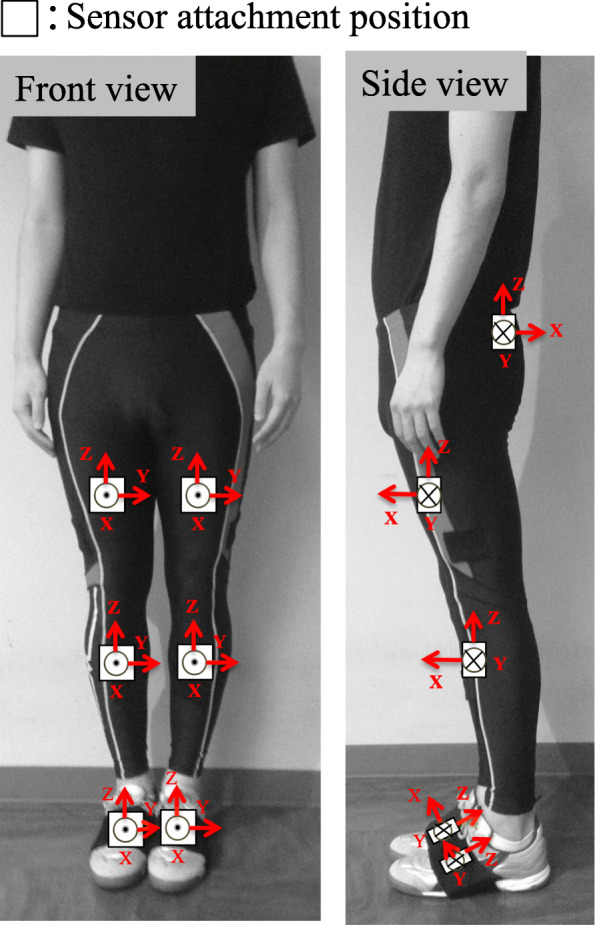
Sensor attachment position on the lower limbs. Seven wearable sensor units are placed on the pelvis, both thighs, both shanks, and both feet of the participants

All data collections were performed on the OA side in the OA group and on the dominant leg in the control group. The dominant side in the control group was defined according to which leg the participants used for kicking. Data were collected using a motion analysis system (H-Gait system, Laboratory of Biomechanical Design, Hokkaido University, Sapporo, Japan) where wearable sensors analyzed the 3-D gait kinematics [8, 12]. Briefly, seven wearable sensor units (TSDN121, ATR-Promotions, Inc., Kyoto, Japan), which consisted of triaxial acceleration and gyro sensors, were placed on seven lower-limb body segments (pelvis, right and left thighs, right and left shanks, and right and left feet), as shown in Fig. 2. Acceleration and angular velocity data were collected simultaneously during gait via wireless connection (Bluetooth) in real time. Sensor specifications were the same as those mentioned in the previous studies [8, 12].

According to a previous study [12], a calibration test for each participant was performed to measure the acceleration data of the sensors in the upright and inclined positions to calculate the initial inclination of each sensor with respect to the gravity. Before each trial, an initial static phase was acquired in the upright position. When the participants started walking, subsequent 3-D orientations from the initial one were estimated by integrating the angular velocity with the drift removal using the MATLAB software (MathWorks, Natick, MA, USA) [13]. The 3-D angular displacement from the initial upright position was calculated in a quaternion according to a previous study [12]. From these data, the spatiotemporal gait parameters; hip joint angles in the sagittal, coronal, and transverse planes; and knee and ankle joint angles in the sagittal plane during walking under each BW condition were evaluated in each participant. This H-Gait system divided 30 s of walking into gait cycles and calculated the angles of each joint for every gait cycle. The median gait cycle represented by this system during 30 s of walking under each BW condition was used for analyses. For the gait cycle, one gait cycle from the heel contact to the next heel contact was normalized to 100%. The swing and stance phases were defined using the heel contact and toe off timings of both legs. The heel contact and toe off timings were detected using the peak angular velocity data of the shank as previously reported [12, 14]. With regard to the validity and reliability of the gait analysis system, Tadano et al. analyzed the kinematics of the lower limbs in walking using the H-Gait system and compared them with that using a camera-based motion analysis system [12]. The correlation coefficients of the hip and knee flexion and ankle dorsiflexion angles were 0.98, 0.97, and 0.78, respectively.

### Statistical analysis

The demographic characteristics and walking speed between the groups were compared using independent Student’s t-tests. One-way ANOVAs with post hoc Bonferroni tests were used to investigate the differences in the NRS pain scores during walking under 100, 75, and 50% BW conditions for the hip OA group. Two-way repeated ANOVAs (3 BW conditions × 2 groups) were performed to assess the main effects of the BW conditions (100, 75, and 50% BW) and groups (control, OA) on the spatiotemporal gait parameters and peak angles of each joint. When the interactions were nonsignificant, the main effects were assessed. If the main effect of the BW condition was statistically significant, post hoc Bonferroni tests were performed to evaluate the significant differences in the spatiotemporal gait parameters and peak angles of each joint among the BW conditions. In addition, the effect sizes for the main effect and interaction between unweighting and group were calculated to determine the magnitude of the differences using eta squared (η^2^). The significance level was set at 0.05. Statistical analyses were performed using the IBM SPSS version 17 software (SPSS Inc., Chicago, IL, USA).

## Results

### Demographic characteristics, walking speed, and pain

Table 1 summarizes the demographic characteristics of the participants and clinical information in the present study. There were no significant differences in age, height, weight, or walking speed between the hip OA and control groups. The hip OA group included three patients with KL grade 3 and 12 with KL grade 4.

**Table 1:** Demography and walking speed

	Hip OA (*n* = 15)	Control (*n* = 15)	*P* value
Age, years	60.4 (9.6)	61.2 (6.3)	0.780
Height, cm	152.8 (2.9)	155.8 (3.7)	0.174
Weight, kg	57.1 (11.4)	53.5 (7.3)	0.329
Walking speed, km/h	1.2 (0.3)	1.3 (0.4)	0.636
OA KL grade 3 (moderate)	3 cases		
OA KL grade 4 (severe)	12 cases		
Harris hip score, point	45.1 (15.3)		

Data are presented as mean (standard deviation). *OA* Osteoarthritis, *KL* Kellgren and Lawrence

**Fig. 3:**
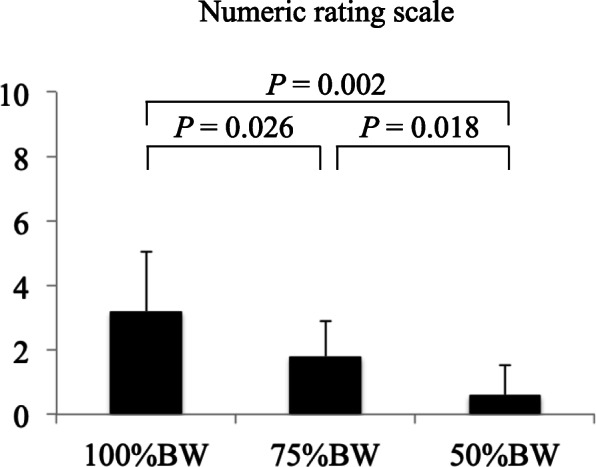
Numeric rating scale pain score under different body weight conditions in the hip osteoarthritis group. The scores 0 and 10 represent no pain and the worst pain, respectively. The bars represent the mean, and the error bars represent the standard deviation. BW, body weight

In the hip OA group, the NRS pain score was significantly lower at 50% BW condition than at the 100% (*P* = 0.002) and 75% (*P* = 0.018) BW conditions. Moreover, the NRS pain score was significantly lower at 75% BW condition than at 100% BW condition (*P* = 0.026) (Fig. 3).

### MDCs on the spatiotemporal gait parameters and the peak hip/knee/ankle joint angles

MDCs on the spatiotemporal gait parameters and the peak hip/knee/ankle joint angles were as follows: 12.1 cm, for the step length; 18.4step/min, for the cadence; 5.1°, for the peak hip flexion angle; 3.4°, peak hip extension angle; 3.9°, peak hip abduction angle; 2.5°, peak hip adduction angle; 3.3°, peak hip external rotation angle; 3.6°, peak hip internal rotation angle; 7.0°, peak knee flexion angle; 3.5°, peak knee extension angle; 5.9°, peak ankle dorsiflexion angle; and 5.2°, peak ankle plantar flexion angle.

### Spatiotemporal gait parameters

**Fig. 4:**
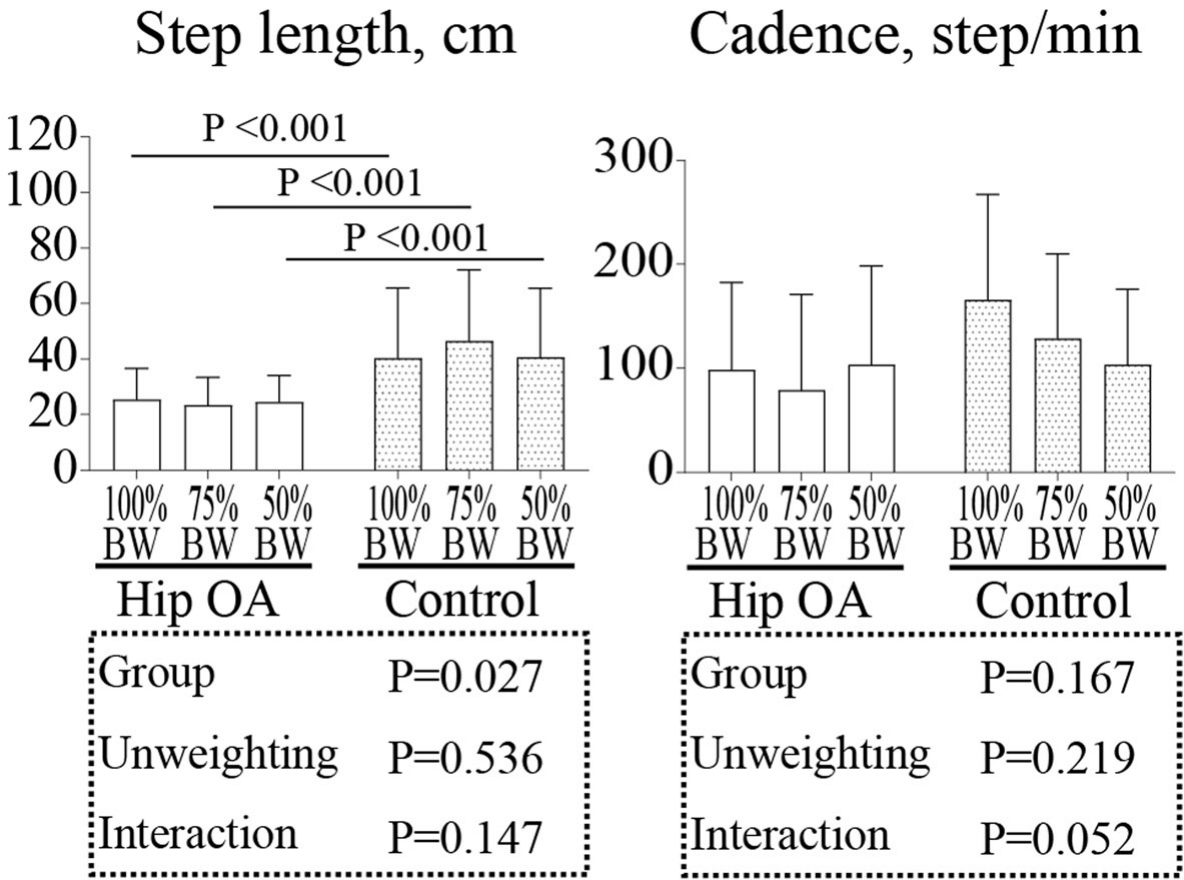
Spatiotemporal gait parameters under different body weight conditions in the hip osteoarthritis and control groups. The bars represent the mean, and the error bars represent the standard deviation. BW, body weight

For the step length, two-way ANOVA showed a statistical difference between the groups (*P* = 0.027) but not between the BW conditions (100, 75, and 50%) (*P* = 0.536). No interaction was detected between the groups and BW conditions (*P* = 0.147) (Table 2). Post hoc Bonferroni tests showed that the step length in all BW conditions in the hip OA group decreased compared with that in the control group (*P* < 0.001). For the cadence, two-way ANOVA did not show a significant difference between the groups (*P* = 0.167) and BW conditions (100, 75, and 50% BW) (*P* = 0.219). No interaction was detected between the groups and BW conditions (*P* = 0.052) (Fig. 4).

**Table 2:** The spatiotemporal gait parameters and peak angles of the hip, knee, and ankle joints

Variables	Group	100% BW	75% BW	50% BW	Effect size (group)	Effect size (unweighting)
Step length, cm	Hip OA	25.3 (14.9–35.6)	23.3 (13.1–33.6)	24.5 (14.5–34.4)	0.83	N/A
	Control	40.3 (20.0–50.7)	46.5 (36.2–56.8)	40.7 (30.7–50.6)		
Hip FLX, degree	Hip OA	22.0 (16.8–27.1)	20.1 (15.2–24.9)	16.2 (11.8–20.6)	N/A	0.05
	Control	25.6 (20.5–30.8)	24.4 (19.6–29.2)	21.0 (16.6–25.4)		
Hip EXT, degree	Hip OA	−0.9 (−4.0–2.3)	0.0 (−2.9–3.0)	− 0.6 (−3.4–2.2)	0.04	N/A
	Control	−4.5 (−7.7−−1.4)	− 3.9 (−6.8−−0.9)	− 4.8 (− 7.6−− 2.0)		
Hip ADD, degree	Hip OA	2.0 (0.2–3.8)	1.7 (0.1–3.4)	0.1 (−1.1–1.4)	N/A	0.01
	Control	4.6 (2.8–6.4)	3.3 (1.7–4.9)	2.3 (0.9–3.8)		
Hip IR, degree	Hip OA	7.4 (4.5–10.3)	6.3 (3.6–9.0)	5.0 (2.7–7.2)	N/A	0.01
	Control	7.4 (4.5–10.3)	5.9 (3.2–8.5)	6.1 (3.8–8.3)		
Knee FLX, degree	Hip OA	47.2 (37.6–56.8)	44.0 (34.1–53.8)	39.3 (30.8–47.8)	N/A	0.11
	Control	59.9 (50.3–69.5)	54.7 (44.9–64.6)	51.9 (43.4–60.4)		
Knee EXT, degree	Hip OA	−7.1 (−9.8−−4.5)	−4.5 (−6.5−−2.5)	−2.8 (−4.8−−1.0)	N/A	0.01
	Control	−3.5 (− 6.2−−0.9)	−3.4 (−5.5−− 1.5)	− 2.3 (−4.2−−0.5)		
Ankle PF, degree	Hip OA	5.5 (−0.1–11.4)	10.0 (3.9–16.3)	11.1 (4.3–17.9)	N/A	0.08
	Control	7.0 (1.2–12.8)	11.4 (5.2–17.6)	14.6 (7.8–21.4)		

Data are presented as mean (95% CI). *OA* Osteoarthritis, *BW* Body weight, *FLX* Flexion, *EXT* Extension, *ADD* Adduction, *IR* Internal rotation, *PF* Plantar flexion

### Effects of unweighting on the peak hip/knee/ankle joint angles

**Fig. 5:**
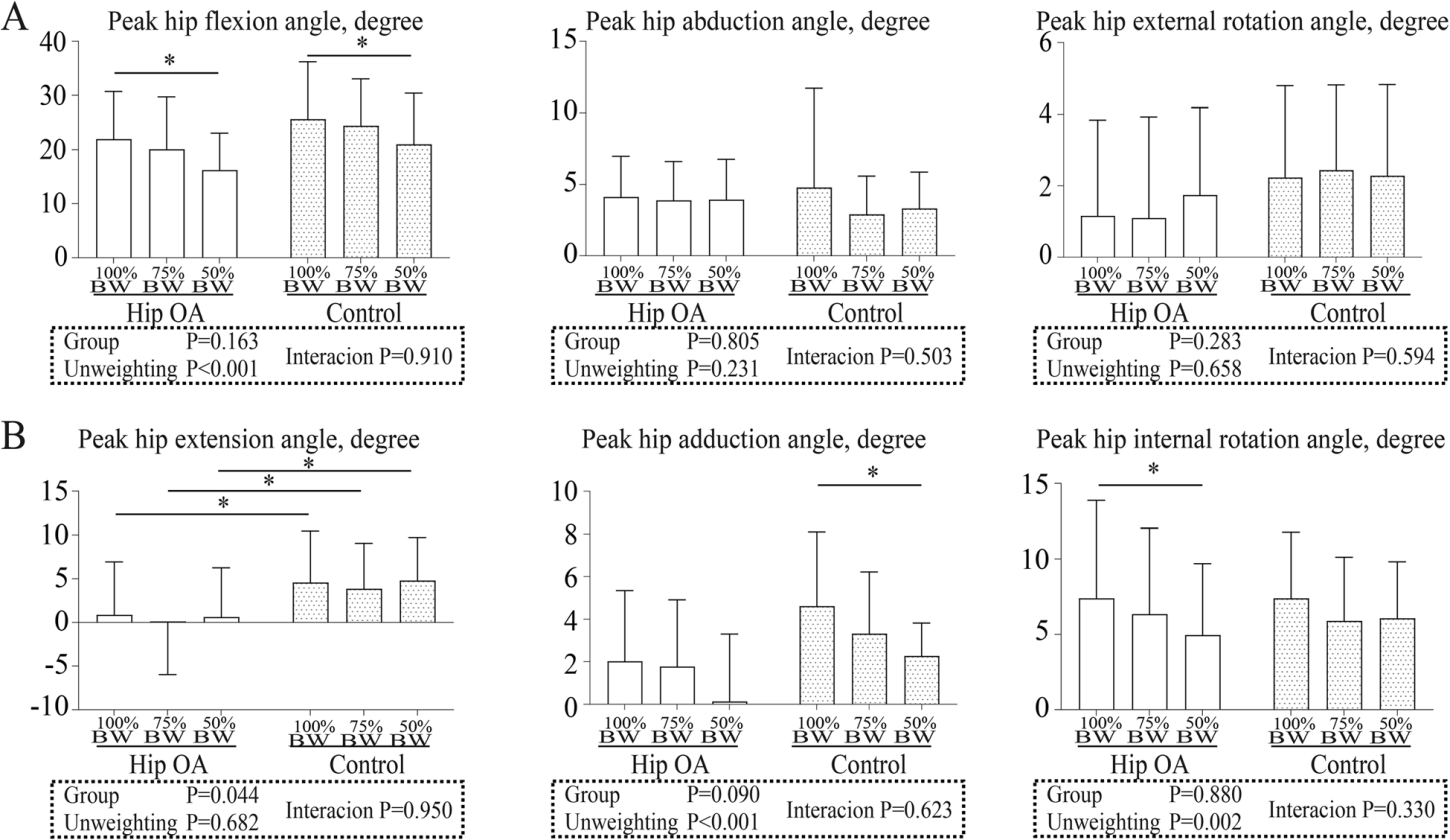
Hip kinematics under different body weight conditions in the hip osteoarthritis and control groups. **a** Swing and **b** stance phases. The bars represent the mean, and the error bars represent the standard deviation. *, *P* < 0.05 with post hoc Bonferroni tests. OA, osteoarthritis; BW, body weight

For the peak hip flexion angle during the swing phase, two-way ANOVA showed a significant difference between the BW conditions (*P* < 0.001) but not between the groups (*P* = 0.163). No interaction was detected between the groups and BW conditions (*P* = 0.910) (Fig. 5a). Post hoc Bonferroni tests showed that the peak hip flexion angle at 50% BW condition in both groups decreased statistically significantly compared with that at 100% BW condition (hip OA, *P* = 0.011; control, *P* = 0.049). For the peak hip abduction and external rotation angles during the stance phase, two-way ANOVA did not show a significant difference between the groups and BW conditions. No interaction was detected between the groups and BW conditions.

For the peak hip extension angle during the stance phase, two-way ANOVA showed a significant difference between the groups (*P* = 0.044) but not between the BW conditions (*P* = 0.682). No interaction was detected between the groups and BW conditions (*P* = 0.950) (Fig. 5b). Post hoc Bonferroni tests showed that the peak hip extension angle in all BW conditions in the hip OA group decreased compared with that in the control group (*P* < 0.001). For the peak hip adduction and internal rotation angles during the swing phase, two-way ANOVA showed significant differences between the BW conditions (adduction, *P* < 0.001; internal rotation, *P* = 0.002) but not between the groups. No interaction was detected between the groups and BW conditions. Post hoc Bonferroni tests showed that the peak hip adduction angle at 50% BW condition in the control group decreased statistically significantly compared with that at 100% BW condition (*P* = 0.012). Post hoc Bonferroni tests showed that the peak hip internal rotation angle at 50% BW condition in the hip OA group decreased statistically significantly compared with that at 100% BW condition (*P* < 0.001).

**Fig. 6:**
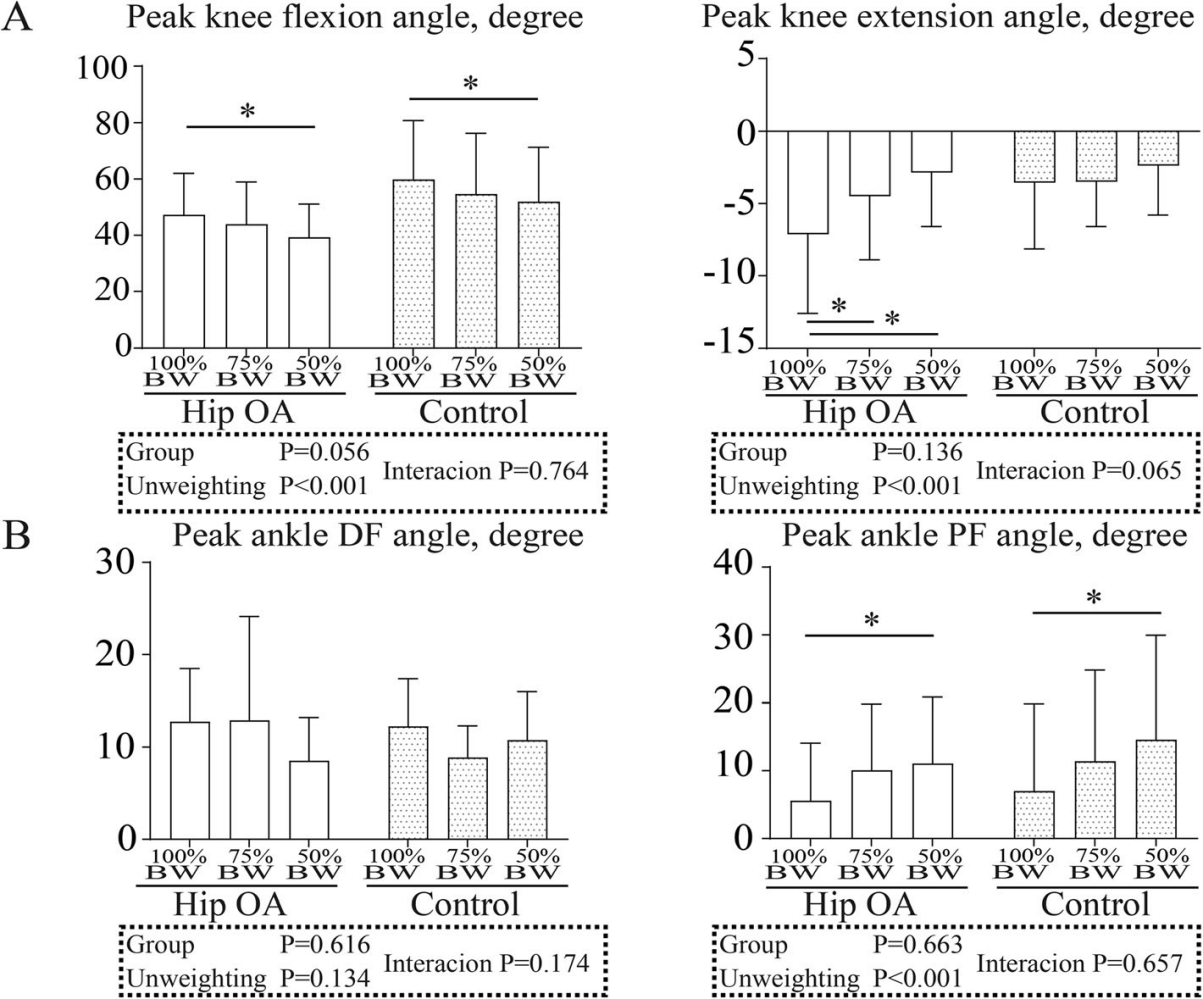
Kinematics of the **a** knee and **b** ankle joints under different body weight. The bars represent the mean, and the error bars represent the standard deviation. *, *P* < 0.05 with post hoc Bonferroni tests. OA, osteoarthritis; BW, body weight; DF, dorsiflexion; PF, plantar flexion

For the peak knee flexion and extension angles, two-way ANOVA showed a significant difference between the BW conditions (*P* < 0.001) but not between the groups. No interaction was detected between the groups and BW conditions (Fig. 6a). Post hoc Bonferroni tests showed that the peak knee flexion angle at 50% BW condition in both groups decreased statistically significantly compared with that at 100% BW condition (hip OA, *P* = 0.002; control, *P* = 0.002) and the peak knee extension angle at 75 and 50% BW conditions in the hip OA group decreased statistically significantly compared with that at 100% BW condition (75% vs. 100% BW, *P* = 0.029; 50% vs. 100% BW, *P* < 0.001).

For the peak ankle plantar flexion angle, two-way ANOVA showed a significant difference between the BW conditions (*P* < 0.001) but not between the groups. No interaction was detected between the groups and BW conditions (Fig. 6b). Post hoc Bonferroni tests showed that the peak ankle plantar flexion angle at 50% BW condition in both groups increased statistically significantly compared with that at 100% BW condition (hip OA, *P* = 0.020; control, *P* = 0.001).

Table 2 summarizes the effect sizes of each parameter that two-way ANOVA showed significant differences in the present study. The effect size of the step length between the groups was large (η^2^=0.83). The effect sizes of the peak knee flexion and ankle plantar flexion angles were medium (η^2^=0.11 and η^2^=0.08). The effect sizes of other parameters were small.

## Discussion

In the present study, we first investigated the NRS pain score and gait kinematics during walking on the LBPP treadmill in the participants with hip OA. As expected, this study showed that unweighting by the LBPP treadmill decreased the NRS pain score among the patients with hip OA. Although the LBPP altered the gait kinematics, there were no significant differences between the hip OA and control groups, suggesting that the LBPP treadmill is desirable for decreasing pain after an aerobic exercise rather than the alteration of gait kinematics among patients with hip OA. This finding is consistent with those of a previous study that used the LBPP treadmill to assess acute knee pain during weight-bearing exercise in a population of overweight patients with knee OA [5]. Because gait impairments due to hip pain lead to decreased endurance and muscle strength in the lower limbs, it is clinically important for these patients to perform gait training under safe and comfortable conditions [3, 7] with less load on the hip joint. To reduce gait alterations during the LBPP training, the 75% BW condition may be useful for participants with hip OA, as this condition significantly reduced pain but did not significantly affect the gait kinematics.

The findings of the present study revealed that contrary to our expectation, unweighting significantly decreased the peak hip and knee flexion angles and increased the peak ankle plantar flexion angle during walking on the LBPP treadmill in both hip OA and control groups. The finding that the peak hip and knee flexion angles decreased during the swing phase under the unweighting condition is consistent with that of previous reports using a treadmill with a harness system [15, 16]. These kinematic changes during gait could be explained by the higher center of gravity due to traction force. Therefore, this study suggests that unloading treadmill walking does not drastically alter the gait kinematics among patients with hip OA and clinicians should consider these unweighting effects on gait kinematics with regard to the use of the LBPP treadmill for patients with hip OA.

The significant difference in the step length between the hip OA and control groups in the present study was large effect size (η^2^=0.83) and may reflect the characteristics of patients with hip OA during walking. More specifically, the shorter step length in the hip OA group than in the control group observed in this study was consistent with that in previous studies that showed that the participants with hip OA walked with 7–10% shorter step length than the age-matched control group [17, 18]. Although the finding of a lower peak extension angle during the stance phase in the hip OA group compared with that in the control group is also consistent with those of previous studies [19, 20], the effect size between groups was small (η^2^=0.04), suggesting that this difference between groups is within the measurement error. Therefore, unweighting by the LBPP treadmill alter the gait kinematics for all participants, not just participants with hip OA. The H-Gait system employs gravity to determine the segment position and orientation. Considering that the LBPP treadmill uses a pressurized chamber to decrease the BW and does not alter gravity, we believe that this H-Gait system could accurately address the kinematics during walking on the LBPP treadmill. However, because this H-Gait system has not yet been validated for use in an altered gravity environment or with the LBPP treadmill, the future study should address sensor adjustment to account for an altered gravity environment.

This study has several limitations. First, only the effects of unweighting on the kinematics of the lower limbs were investigated, rather than including the effects of unweighting on the kinematics of the trunk and upper limbs. Second, this system may have a larger measurement error compared with a camera-based system such as a Vicon system. However, we believed that wearable sensors are an excellent application for this investigation as the treadmill design makes it difficult to acquire motion analysis data using traditional skin marker motion analysis technologies. Third, the present study targeted participants with the moderate or severe hip OA that will be received THA within 3 months of data collection; therefore, unweighting during gait could affect hip pain differently for participants with early stage hip OA. Future study should investigate the training effect of LBPP among patients with early stage hip OA.

## Conclusions

Unweighting by the LBPP treadmill decreased pain in the hip OA group but did not drastically alter the gait kinematics compared with that in the control. Therefore, regarding the use of the LBPP treadmill for patients with hip OA, clinicians should consider the benefits of pain reduction rather than the kinematic changes.

## Data Availability

The datasets used and/or analyzed during the current study available from the corresponding author on reasonable request.

## Abbreviations

3-D: Three-dimensional
BW: Body weight
KL: Kellgren and Lawrence
LBPP: Lower-body positive pressure
NRS: Numeric rating scale
OA: Osteoarthritis
MDCs: Minimal detectable changes
THA: Total hip arthroplasty
